# Supplementary material for: Geometric and electronic structure probed along the isomerisation coordinate of a photoactive yellow protein chromophore
Source: Nat Commun. 2020 Jun 4;11:2827. doi: 10.1038/s41467-020-16667-x (PMC7272410; doi:10.1038/s41467-020-16667-x)
Supplement: Supplementary file 1 — Supplementary Information [file 41467_2020_16667_MOESM1_ESM.pdf]

Supplementary Materials for:

Geometric and electronic structure probed along the isomerisation coordinate of a photoactive yellow protein chromophore

Cate S. Anstöter, Basile F. E. Curchod and Jan R. R. Verlet

Department of Chemistry, Durham University, Durham DH1 3LE, United Kingdom

Supplementary Figure 1

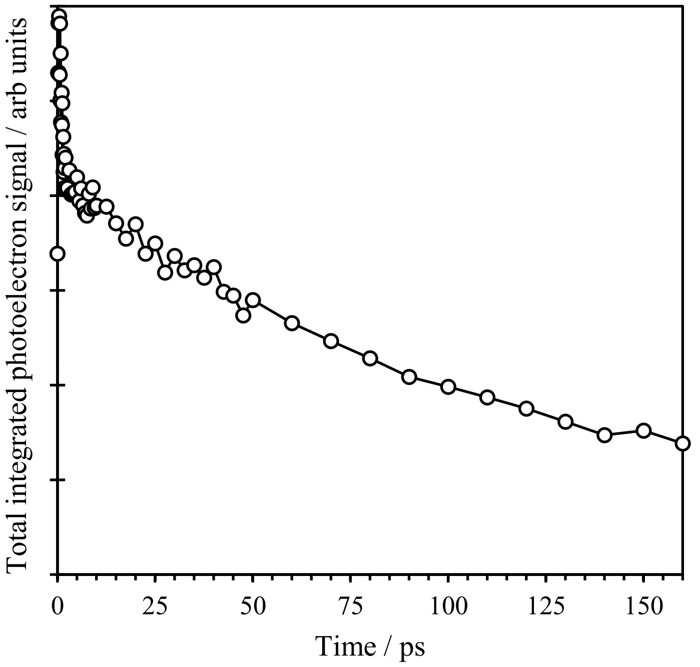

Integrated pump-probe photoelectron signal over longer timescales than shown in main text.

Supplementary Note 2

Photoelectron spectra of *p*CK$^-$ were acquired using a nanosecond Nd:YAG pumped OPO operating at $h\nu$ = 3.45 and 4.20 eV (Figure S1). Pulses had a duration of ~5 ns and pulse energies were kept low so that the signals arose solely from single-photon processes. The photon energy $h\nu$ = 3.45 eV was chosen specifically as in this energy range, excitation is the least resonant with excited states of the anion that could potentially distort the photoelectron spectrum. From these measurements, the vertical detachment energy (VDE) and adiabatic detachment energy (ADE) can be determined as shown in Supplementary Figure 1.

Supplementary Figure 2

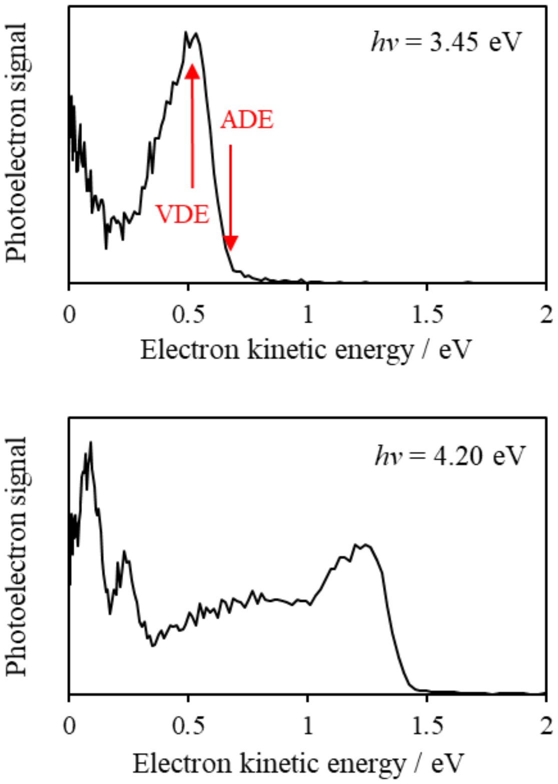

Photoelectron spectra of *p*CK$^-$ taken at two photon energies using nanosecond light. The adiabatic detachment energy (ADE) and vertical detachment energy (VDE) are indicated in the $h\nu$ = 3.45 eV spectrum which was chosen because at this energy, there are the fewest excited states accessed. The $h\nu$ = 4.20 eV spectrum highlights the contribution from the D_1 excited state of the neutral.

Supplementary Note 3

Changes of the β_4 parameter as a function of both the kinetic energy and time are plotted in Supplementary Figure 2 and should be taken together with Figure 1c in the main text, which shows the corresponding β_2 parameters. Overall, the β_4 parameters are effectively zero across all times and all energies and therefore suggest that initial alignment is not a major factor in the determination of the PADs using only the β_2 parameter.

Supplementary Figure 3

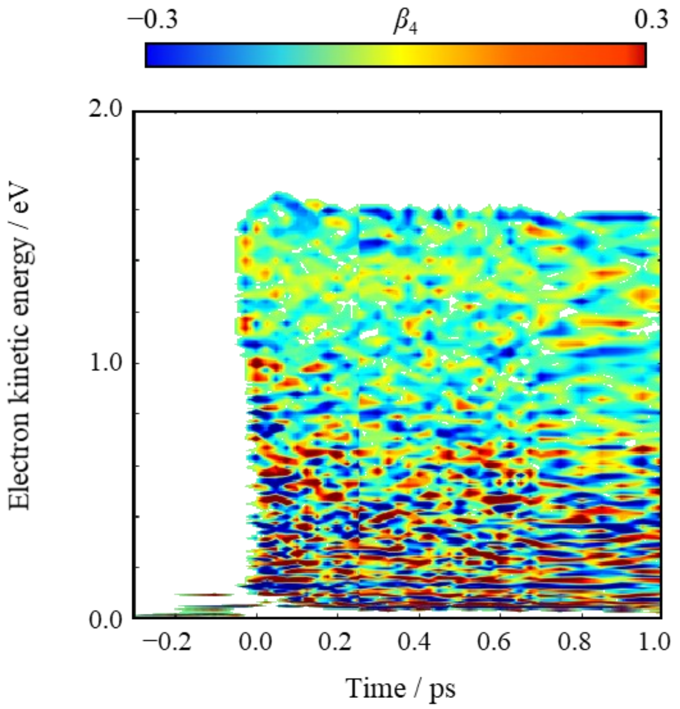

Time-resolved β_4 false colour map, where values are omitted whenever the signal level is <0.1 in Figure 1**b** of main text. The corresponding time-resolved β_4 false colour map is shown in Figure 1**c** of the main text.

Supplementary Figure 4

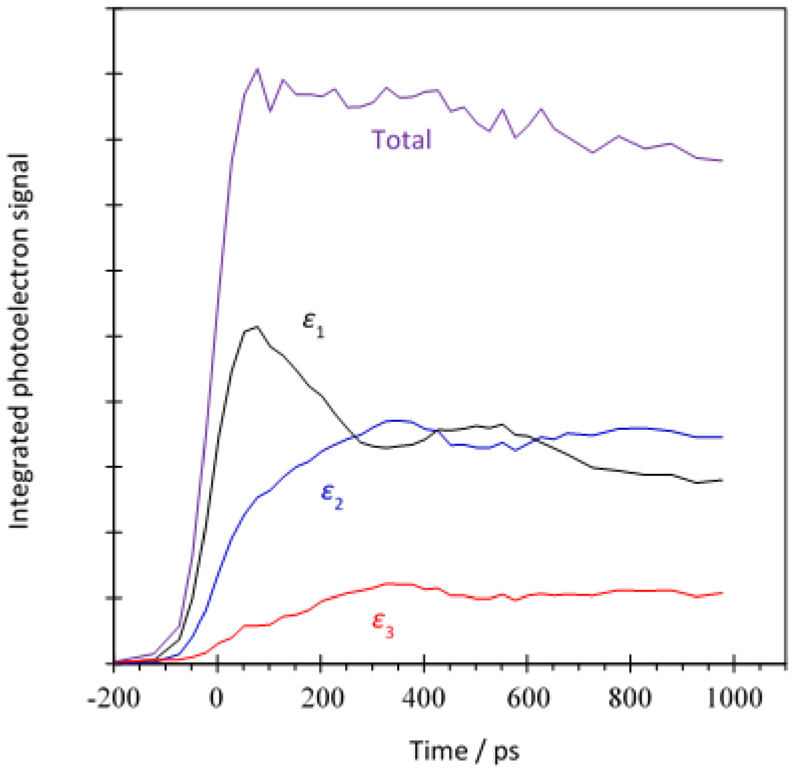

Integrated pump-probe photoelectron signal: total (purple), ε_1 (black), ε_2 (blue), and ε_3 (red), as defined in the main manuscript, Figure 1.

Supplementary Methods

Supplementary Figure 3(a) shows a typical photoelectron image acquired in the pump-probe experiment (t = 50 fs). Supplementary Figure 3(b) shows the pump-probe signal more clearly by saturating the central peak which arises from the thermionic emission channel. The analysis of the image uses polar onion peeling, one of many programs that reconstructs the 3D velocity vector distribution from the 2D photoelectron image shown in Figure S3(a) and (b). It starts by converting the image from Cartesian to polar coordinates. At a large radius, r, the signal as a function of polar angle θ, is fit to the function

$$I(\theta; r) = \sigma/4\pi\ [1 + \beta_2 P_2(\cos\theta) + \beta_4 P_4(\cos\theta)],$$

where $I(\theta; r)$ is the photoelectron signal as a function of the angle, θ, between the laser polarisation axis and the photoelectron emission velocity vector, σ is the detachment cross-section, β_2, and β_4 are anisotropy parameters, and $P_2(\cos\theta)$ and $P_4(\cos\theta)$ are the second- and fourth-order Legendre polynomials. Using the fit parameters σ, β_2, and β_4, a 2D photoelectron image is generated and subtracted from the experimental image. The same procedure is then repeated for the next smaller radius until the centre is reached. σ, provides the spectrum as a function of r which is proportional to the velocity. This is shown in Supplementary Figure 3(c), while the corresponding β_2 and β_4 spectra (as a function of r) are shown in Supplementary Figures 3(d) and (e). Conversion to energy space requires division by the appropriate Jacobian (r) and calibration, which is done using the photoelectron image of iodide.

All raw photoelectron images are freely available. The time-resolved spectra and images have an average of the spectra before $t = 0$ removed ($t = -325$, -225, and -125 fs). Images were reconstructed using poplar onion peeling with a centre (251, 247).

Supplementary Figure 5

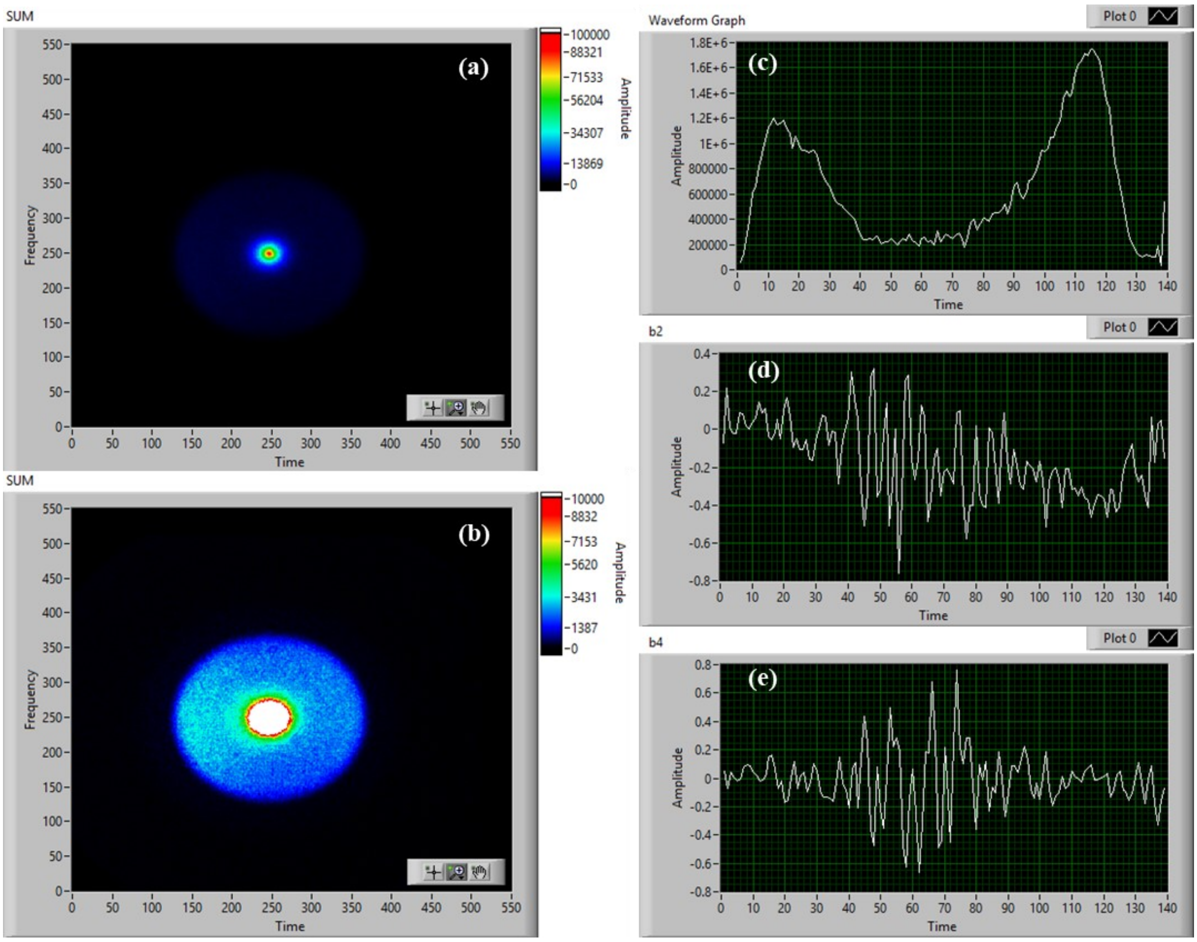

Overview of data analysis. (a) Raw photoelectron image taken with a 444 nm pump and 800 nm robe with a time delay of 50 fs. (b) Same as (a) but /10 scale to emphasis pump-probe signal at larger radius, *r*. Result from poplar onion peeling algorithm to yield: (c) photoelectron spectrum as a function of *r* (proportional to electron speed); (d) β_2 spectrum as a function of *r*; and (e) β_4 spectrum as a function of *r*.

Supplementary Figure 6

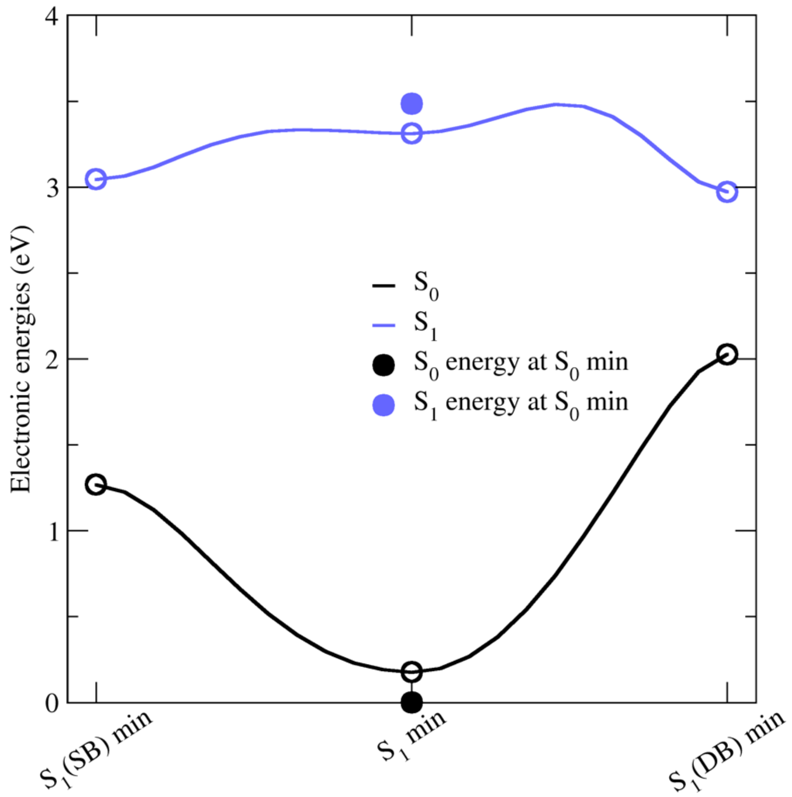

LIIC pathways at the SA2-CASSCF(12,11)/6-31G* level of theory. The pathways were interpolated between the following critical points: S_1 planar minimum to S_1(DB) min, and S_1 planar minimum to S_1(SB) min.

Supplementary Table 1

S_1 min planar ($E(S_1)$ = -533.64953888 h)

C	2.7742787206	1.1288470250	0.0067516867
C	1.4131659610	1.3699829973	0.0352968245
C	0.4535918154	0.3151715807	0.0119461018
C	0.9724712067	-1.0191388973	-0.0429407395
C	2.3365872528	-1.2751235473	-0.0720871584
C	3.3201422028	-0.2192306601	-0.0491354638
H	3.4790595544	1.9420179399	0.0252580040
H	1.0567679374	2.3857264160	0.0766012785
H	0.2914387255	-1.8485882166	-0.0620410201
H	2.7019596051	-2.2866791383	-0.1133248884
C	-0.9653116169	0.6256099842	0.0437679736
H	-1.2490300451	1.6605403063	0.0862980584
C	-2.0056045562	-0.3353344446	0.0246141054
H	-1.7694169551	-1.3819071386	-0.0183522285
C	-3.3850010730	0.0298080551	0.0595194047
C	-4.4241060907	-1.0849721424	0.0342495816
H	-5.0513440805	-1.0283158944	0.9214042894
H	-5.0802064399	-0.9617075789	-0.8248124833
H	-3.9843506219	-2.0767821280	-0.0108163845
O	4.5469065978	-0.4261865165	-0.0745827693
O	-3.8010841000	1.1994119986	0.1095538273

S_1(DB) min ($E(S_1)$ = -533.66199627 h)

C	2.9043379494	-0.3192868693	1.0375667181
C	1.6226773512	-0.4171063611	1.5203419986
C	0.4649311877	-0.3470074461	0.6806529544
C	0.7469190736	-0.1718429719	-0.7102266332
C	2.0280560041	-0.0725629232	-1.2037561129
C	3.2009945780	-0.1367140115	-0.3661341988
H	3.7431583574	-0.3764317506	1.7127137008
H	1.4729704338	-0.5522041203	2.5816008384
H	-0.0759742271	-0.1145993626	-1.4047721059
H	2.1892627818	0.0603757812	-2.2616042821
C	-0.8320156004	-0.4346156021	1.2199367900
H	-0.9376228111	-0.5168400712	2.2904984965
C	-2.0660484466	-0.2951242770	0.4490775802
H	-2.4929401038	0.6859707761	0.2849555446
C	-2.8168674359	-1.3947608502	-0.1342789577
C	-4.0342115629	-1.0371757081	-0.9737874602
H	-4.5257892811	-1.9428032220	-1.3057412192
H	-4.7398735623	-0.4387766028	-0.4036627235
H	-3.7439666654	-0.4542810799	-1.8435204430
O	4.3616868557	-0.0416411830	-0.8070347629

O	-2.5183145455	-2.5693072102	0.0274934455

S_1(SB) min ($E(S_1)$ = -533.65934428 h)

C	2.4720421848	0.9437934043	-0.7830170302
C	1.1500618374	1.0797365103	-0.4359519843
C	0.4722137063	0.1443665759	0.4075380298
C	1.2453812503	-0.9647805663	0.8755430639
C	2.5687394129	-1.1366489440	0.5505191680
C	3.2601715955	-0.1812134835	-0.3023194876
H	2.9691713004	1.6554765436	-1.4178430720
H	0.5843492568	1.9162820589	-0.8037178872
H	0.7517009691	-1.6830336731	1.5043844833
H	3.1382237228	-1.9752088557	0.9098990057
C	-0.9436008113	0.3146144766	0.7758142946
H	-1.1668772696	0.8755600795	1.6666670386
C	-2.0004979976	-0.2230070203	0.0192827370
H	-1.7558907120	-0.7862849041	-0.8682107701
C	-3.3616571877	-0.0786442260	0.3470764713
C	-4.3863263071	-0.7314298560	-0.5831236559
H	-4.9933389762	-1.4448566596	-0.0273188845
H	-5.0696344268	0.0219439029	-0.9727655636
H	-3.9362140689	-1.2508901510	-1.4251737780
O	4.4505777820	-0.3179529159	-0.6018500986
O	-3.8176812613	0.5353277034	1.3317359200
